# Silver-Exchanged Zeolites: Preparation and Applications—A Review

**DOI:** 10.3390/ma18204779

**Published:** 2025-10-19

**Authors:** Marin Senila, Eniko Kovacs, Lacrimioara Senila

**Affiliations:** INCDO-INOE 2000, Research Institute for Analytical Instrumentation, 67 Donath Street, 400293 Cluj-Napoca, Romania; eniko.kovacs@icia.ro

**Keywords:** zeolite modification, natural zeolite, synthetic zeolite, zeolite characteristics, antimicrobial activity, water treatment, environmental applications

## Abstract

Zeolites are widely acknowledged as minerals with outstanding characteristics, primarily due to their complex porous structure and large specific surface area. The modification of natural and synthetic zeolites can improve their properties, making them suitable for a wider range of applications. In recent years, silver-exchanged natural zeolites have been increasingly studied because silver is known for its antimicrobial and electrical conductivity properties, which enhance their utility in various fields. This study aims to provide a comprehensive review specifically focused on the silver-zeolite composite system. It synthesizes advancements in silver modified zeolites, offering a targeted analysis that connects synthesis methodologies to enhanced properties and applications. The paper is structured to include an overview of the general characteristics of both natural and synthetic zeolites, and methods for their modification to serve as supports for Ag^+^ and silver nanoparticles (AgNPs). It subsequently covers the properties of silver-exchanged zeolites and their principal applications. The study also summarizes the advantages and limitations of these materials, along with an analysis of future trends regarding new production possibilities and potential applications.

## 1. Introduction

Zeolites are aluminosilicate minerals characterized by a three-dimensional open framework, featuring a complex porous structure and a high specific surface area [1,2]. Natural zeolites were discovered in 1756 by a Swedish chemist, Axel Fredrik Cronstedt, the founder of modern mineralogy. He named these minerals from the Greek words zeo (‘to boil’) and lithos (‘stone’) based on their characteristic behavior of releasing moistness on their surface upon heating [3,4,5]. For nearly two centuries, the practical application of zeolites was not thoroughly investigated. However, the discovery of their unique features generated significant interest. Consequently, there is an increasing focus on the study of their physical, chemical, and mineralogical characteristics, as well as on the identification of potential applications for these compelling materials [6].

Natural zeolites contain as primary building units tetrahedra of silicon and aluminum oxides which are unified by oxygen atoms into infinite two-dimensional and three-dimensional secondary building units [4,7]. The presence of aluminum in this network assigns an overall negative charge to the zeolite structure. Therefore, the Si/Al ratio plays a significant role in determining the zeolite’s characteristics. The negative framework charge is compensated by hydrated alkaline cations, which are commonly accompanied by water molecules within the zeolite channels [8,9,10]. The physical, chemical, and mineralogical characteristics of natural zeolites enable them to participate in ion-exchange processes and to act as molecular sieves for separating different substances. Thus, zeolites are inexpensive and environmentally friendly adsorbent materials.

The genesis of natural zeolite species depends on the class of minerals they contain [11]. Natural zeolites of hydrothermal origin are characterized by very pure, euhedral crystals, but these crystals are only a few centimeters in size, limiting their practical applications. In contrast, natural zeolites of sedimentary origin, which form in volcaniclastic rocks at lower temperatures and through slower processes, are more widespread and have practical uses. These zeolites often co-crystallize with other minerals and usually contain impurities, sometimes up to tens of percent [6].

To expand the potential applications of zeolites, manufacturing synthetic zeolites became necessary. This necessity arises primarily due to the presence of impurities in natural zeolites, which limit their adsorption capacity. The advantages of synthetic zeolites extend far beyond the mere absence of mineralogical impurities. While natural zeolites are limited to a small number of framework topologies—only a few of which occur in geological deposits—more than 250 distinct zeolite structures have been identified and synthesized to date. This structural diversity is achieved almost exclusively through laboratory synthesis. Furthermore, synthetic zeolites allow for the stabilization of frameworks across a much broader range of Si/Al ratios compared to their natural counterparts, enabling precise tuning of physicochemical properties such as acidity, hydrophobicity, and thermal stability. This expanded compositional flexibility makes synthetic zeolites highly versatile materials for catalytic, sorption, and separation processes [12].

Among the most well-known synthetic zeolites are types A, X, Y, and zeolite Socony mobil-5 (ZSM-5). A key advantage of synthetic zeolites lies in the fact that they can be tailored for different applications [3,13].

Despite their excellent properties, both natural and synthetic zeolites may have limited capabilities for certain applications. For this reason, extensive research has been conducted to modify zeolites to render them suitable for a wider range of uses. Silver (Ag) is well known for its antimicrobial and electrical conductivity properties, making it valuable across a range of applications. Commonly, Ag is incorporated into a support material that controls its release. Ag-modified zeolites enhance the sorption and photocatalytic properties of zeolites [14].

Numerous research papers have been published on the modification of natural and synthetic zeolites with silver and their applications. However, to the best of our knowledge, no comprehensive review has systematically detailed the methods of silver incorporation into zeolites and the resulting applications. This review aims to provide a comprehensive overview of recent advancements in the modification of natural and synthetic zeolites with silver. The focus is on integrating silver ions (Ag^+^) and silver nanoparticles (AgNP) into their structure, and their applications in antimicrobial activity, water treatment, and gas separation. The paper is structured to encompass a concise overview of the general characteristics of zeolites, methods for modifying zeolites to serve as supports for Ag^+^ and AgNP, the properties of silver-exchanged zeolites, and their primary applications. Additionally, trends and future research directions regarding the use of silver-exchanged zeolites are discussed.

## 2. Materials and Methods

To identify relevant publications related to silver-exchanged zeolites, their preparation and applications, a search was conducted using the Clarivate database. The keywords ‘Zeolite’ AND ‘Silver’ AND ‘Modified’ were used in the initial search. All these documents were evaluated, and only those focusing on the modification of zeolites with silver for subsequent use in water treatment and gas separation were included in this review.

## 3. Zeolites—General Characteristics

**Figure 1:**
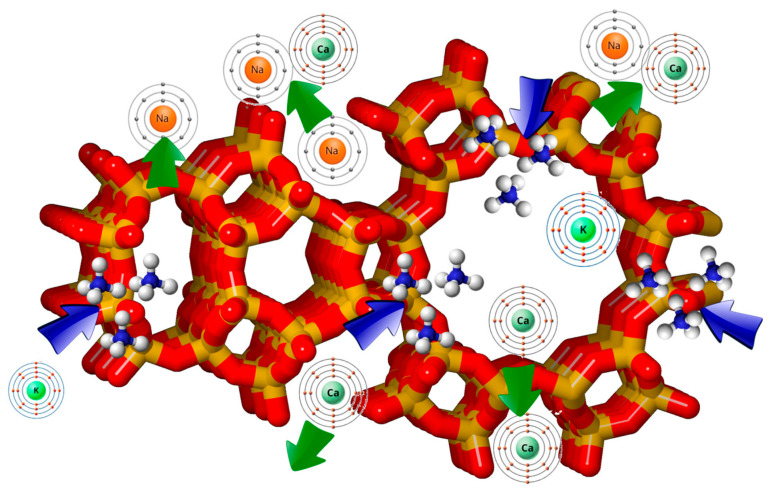
Zeolite structure. Reproduced from Mancinelli et al. [18]. (green arrows—sodium and calcium, blue—potassium).

More than 60 types of natural zeolite have been discovered around the world, but only six occur in significant quantities in natural deposits: analcime, chabazite, clinoptilolite (a class of the heulandite group), erionite, mordenite, and phillipsite [4,9]. The structure and chemical formulas of several natural zeolites is presented in Figure 1.

Zeolite deposits are widespread around the world, and zeolite markets have developed on all continents. Large sedimentary deposits of natural zeolites are found in countries such as Australia, USA, Cuba, Canada, Mexico, Turkey, Bulgaria, Romania, Hungary, Serbia, Slovakia, Italy, Russia, Ukraine, Georgia, or Japan [10].

The general formula of natural zeolite is (M_x/n_) [(AlO_2_)_x_ (SiO_2_)_y_] × wH_2_O, where M is the cation of valence n, the sum of y and x is the total number of tetrahedra in the unit cell, w is the number of water molecules, and the ratio y/x varies from 1 to ꝏ based on the structure [15]. The crystallinity of the zeolite is ensured by the TO_4_ tetrahedron, in which T stands for trivalent (Al, Ga, B), tetravalent (Si, Ge), or pentavalent (P) atoms [16]. These atoms [T] are interconnected via a shared oxygen atom and represent the primary structural unit of the zeolite. These units, tetraders, generate two- and three-dimensional secondary structural components, creating a three-dimensional spatial lattice structure with pores and cavities that are specific for zeolites [17].

The SiO_4_ (and AlO_4_) tetrahedra unify into secondary building units (SBUs) like 4-rings, 6-rings, double 4-rings, etc., which connect to form the “zeolitic pores”. Clinoptilolite has 10-membered and 8-membered ring channels, mordenite has 12-membered ring channels, while chabazite has cage-like structures [19].

The difference in the electronegativity of the [SiO_4_]^4−^ and [AlO_4_]^5−^ tetrahedra results in an excess of a negative charge on the overall zeolite framework. This negative charge is neutralized by cations of alkaline and alkaline earth metals (mainly K^+^, Na^+^, Ca^2+^, Mg^2+^) found in channels and pores of zeolites, concurrently with H_2_O molecules (Figure 1). These cations of alkaline and alkaline earth elements are weakly bound to the zeolite structure, thus can be easily replaced by other cations. For this reason, they are termed interchangeable cations [15]. The Si/Al ratio oversees hydrophobicity, acidity, and stability of zeolites. Zeolites with high Si/Al are more hydrophobic and thermally stable, whereas zeolites with a low Si/Al have a higher cation exchange capacity and stronger interaction with polar molecules [20,21]. Representative structures and their specific formulas are listed in Table 1.

The presence of channels and cavities in the skeletal constitution of zeolites gives them a number of unique physicochemical properties, rendering them versatile materials suitable for a wide range of applications. Zeolites have surface-active centers of acid–base or oxidation–reduction character. These are responsible for their exceptional adsorption and catalytic activity.

Zeolites possess numerous properties, each exhibiting selectivity, high molecular stability, regenerability, and different sorption characteristics [6]. The properties of zeolites are influenced by several factors, including crystal structure, size, density, and anion charge. Natural zeolites have high chemical stability, a large specific surface area and low cost [23]. Zeolites are used in environmental protection (heavy metal, water decontamination, flue gas treatment), agriculture (odor adsorber, fertilizer, feed additive, aquaculture), health and hygiene (pharmaceuticals, cosmetics, detergent additive), water adsorption (desiccant, heat storage), petrochemistry (heterogenous catalysis, purification of gases, extraction of petroleum products), active fillers (construction materials, paper, polymers), etc. [24]. Table 2 presents a comparative overview of the molecular formulas, structural features and physico-chemical properties of synthetic and natural zeolites.

In this context, the current review was conducted to systematically present the latest progress in the treatment and modification of natural and synthetic zeolites, with a focus on their application in the remediation of water and soil contaminated by heavy metals. This work also calls for greater involvement of scientists in advancing research on the use of natural zeolites for the removal of heavy metals from polluted environments, essential for the continuous development of natural zeolites and their applications. Overall, this review contributes to a comprehensive understanding of the treatment and modification of natural zeolite that enhance their performance and applicability.

The production of synthetic zeolites is increasing due to their growing potential and widespread use. Synthetic zeolites can be produced according to the desired structure and without the impurities found in natural zeolites. Common types of synthetic zeolites include A, X, Y, and ZSM-5 [3]. Table 3 summarizes the comparative characteristics of natural and synthetic zeolites, highlighting differences in their composition, structure and main applications.

Synthetic zeolites are used across various fields, including catalysis, detergent, adsorption and separation and environmental uses. They are more expensive than natural zeolites, but their efficiency is much higher due to the high purity. Synthetic zeolites are produced from various silica carriers such as clay minerals (kaolin, halloysite, volcanic glasses, pumice or diatomite), using methods involving hydrothermal synthesis (alkali fusion, alkaline activation, Molten salt method), sol–gel method, microwave assisted synthesis, vapor phase transport method and other green/alternative methods [25].

## 4. Preparation of Silver Exchanged Zeolites

Silver is used across diverse sectors, including medical devices, textiles, cosmetics, paints and water purification systems, due to its functional properties. Silver is also known as being an antibacterial agent used for wastewater purification. Generally, silver is incorporated into an inert support material to control its release. Zeolite is an ideal support material due to its unreactive nature and excellent silver ion-exchange properties [26,27]. The preparation of silver-impregnated zeolite is typically achieved through ion exchange (Ag^+^ replaces Na^+^, K^+^, and Ca^2+^ ions in the zeolite) [28], wet impregnation (which involves soaking the zeolite in an aqueous AgNO_3_ solution, followed by drying to stabilize the material) [29], and chemical reduction (forming Ag nanoparticles inside the zeolite channels) [30].

Silver plays several important roles in zeolites. It provides antibacterial [31] and antifungal properties [32] by gradually releasing Ag^+^ ions that destroy the cell membranes of microorganisms [33]. It also acts as a catalyst [34], Ag-impregnated zeolites can catalyze oxidation [35], reduction [27], or decomposition reactions of pollutants [36,37]. Silver also functions as a filtration material [38,39], as these zeolites are used to purify water and air by inhibiting bacterial growth. Additionally, silver offers thermal and chemical stability, zeolite providing a stable support for Ag nanoparticles, preventing agglomeration and loss of efficiency [40].

For the application of Ag-modified zeolites, the following aspects should be considered: (i) the binding site of the silver ion on the aluminosilicate framework and the potential formation of clusters, and (ii) the structural response of the silver-modified zeolite to increasing temperatures, as most of these materials are used after thermal treatment [14].

The distribution and chemical state of silver in Ag-exchanged aluminosilicate frameworks are strongly influenced by temperature, pretreatment, and ion-exchange variables such as the Ag^+^ source, concentration, pH, contact time, and number of exchange cycles. Additionally, the identity and preconditioning of the parent cation, as well as subsequent pretreatment conditions—including temperature and heating rate—play significant roles. Low-temperature, mild ion-exchange conditions (dilute Ag^+^ solution, moderate contact time, and lower temperatures) and Na-rich parent forms generally favor the stabilization of isolated Ag^+^ cations at both framework and extra-framework exchange sites. In contrast, aggressive exchange conditions (high Ag^+^ concentration and prolonged contact times) or exchange into H-forms promote partial reduction and increased mobility of silver. Thermal pretreatment at moderate temperatures (100–200 °C) induces the formation of Ag_n_ clusters while higher temperatures (300–400 °C) favor growth and sintering into larger Ag^0^ nanoparticles [41,42].

Recently, Gicheva et al. [28] reported the production of silver nanoparticles using several methods, including thermal reduction of Ag exchanged zeolite, chemical reduction by using sodium citrate reduction in aqueous solution and sodium borohydride reduction. The silver nanoparticles obtained were tested for antibacterial activities. Faryad et al. [43] reported the synthesis of solid-state Ag-exchange zeolite through the ion exchange process of zeolite with silver sulfate and silver nitrate salts. The new materials obtained were used for their antimicrobial effect on the elimination of *Escherichia coli* and Gram-positive *Staphylococcus aureus*, which are common bacteria found in wastewater.

The functionalization process using tannic acid as a reducing agent to introduce silver nanoparticles into zeolite was reported by Gattucci et al. [44] This method was carried out at room temperature, in contrast to conventional methods that require heat treatment or chemicals. It leads to uniform dispersion of silver nanoparticles, and its antibacterial activity was tested against both Gram-positive and Gram-negative bacteria [44]. Reske et al. [45] reported on the preparation of an adsorbent composed of zeolite-impregnated graphite oxide for the continuous removal of silver ions (Ag(I)) under elevated temperature and pressure in a fixed-bed column. The study tested temperatures ranging from 100 to 200 °C and pressures from 5 to 25 MPa, with an optimal adsorption achieved at 200 °C and 25 MPa [45]. This material combines zeolite Linde Type A with graphite oxide. Fernandes Rocha et al. [26] developed a new zeolite-based adsorbent modified with silver nanoparticles for the removal of *Microcystis aeruginosa*, achieving 76% efficiency. This cyanobacterium remains stable in water due to its strong hydrophilicity, low specific gravity, and negative charge. Recently, various methods have been employed for its elimination, including ultrasound, chemical algicides, photocatalysis, coagulation-flocculation, air flotation, and ultrasonic waves, often using adsorbents such as activated carbon, clay minerals, biomaterials, and zeolites [26].

Silver-modified clinoptilolite stabilizes various silver species (ions, clusters, nanoparticles) that provide potent and durable antibacterial effects, particularly against *E. coli* and *S. aureus*. Preparation methods (temperature, pretreatment, ion-exchange conditions) influence silver speciation, ion mobility, and thus antimicrobial performance. In addition to disinfection, Ag–clinoptilolite exhibits strong heavy-metal adsorption, making it a multifunctional material for water treatment [46]. Cametti et al. [14] reported the preparation of silver modified zeolite with stilbite (STI) framework type. Through the complete exchange of extra framework ions with Ag^+^ at room temperature, the structure undergoes distortion, resulting in a reduction in symmetry from orthorhombic FM mm to monoclinic F2/m. The Ag^+^ ions are highly disordered, with occupancies ranging from 0.02 to 0.24, and are located within the zeolite cavities. The water molecules bound to Ag^+^ are gradually lost through dehydration, causing the zeolite structure to shrink as the cell volume decreases. At temperatures above 400 °C, the zeolites remain stable but exhibit a more compact framework. The Ag^+^ ions do not reduce to metallic Ag^0^. This study demonstrates that Ag^+^-modified zeolite is thermally stable; although its structure changes during dehydration, the Ag^+^ ions remain within the zeolite and can perform antibacterial and catalytic functions. Specifically, Ag ions can destroy bacteria or catalyze chemical reactions such as oxidation or reduction [14]. Znak et al. [47] reported different methods used for the activation of zeolite, including HCl, H_2_SO_4_, NaOH, NH_4_Cl, and thermal treatment prior to the sorption of silver ions. The best results for silver sorption were obtained with zeolite activated by NaOH. Activation of zeolite significantly enhances its sorption capacity for silver ions compared to non-activated zeolite [47].

Dong et al. [48] reported the preparation of zeolite nanocrystals by chemical reduction under microwave irradiation. The incorporation of silver nanoparticles (AgNPs) into zeolitic frameworks via chemical reduction represents an effective strategy for producing stable, size-controlled nanocomposites. Initially, Ag^+^ ions are introduced into the zeolite channels through ion-exchange processes, after which chemical reducing agents such as sodium borohydride or sodium citrate are employed to achieve in situ reduction to metallic Ag^0^. Confinement within the zeolite micropores restricts nanoparticle agglomeration, yielding well-dispersed AgNPs with narrow size distributions. The resulting Ag–zeolite composites integrate the high surface area, adsorption, and ion-exchange properties of zeolites with the catalytic, electronic, and antimicrobial functionalities of silver. These hybrid materials demonstrate significant potential for applications in heterogeneous catalysis, environmental remediation, gas separation, and biomedical systems [48].

Zeolites’ characteristics are typically determined to assess their composition, mineralogy and structure, morphology, thermal stability, and other physicochemical properties. In the determination of chemical composition are measured major and trace elements, as well as the exchangeable cations. Major elements (Si, Al, Na, K, Ca, Mg, Fe, Mn, P, etc.) in zeolites can be measured using stationary or portable X-ray Fluorescence (XRF), or using a spectrometric method such as atomic absorption spectroscopy (AAS), or inductively coupled plasma-based methods (optical emission spectrometry—ICP-OES, or mass spectrometry—ICP-MS), following acid digestion. However, Si is very difficult to dissolve, thus its determination using an atomic spectrometric method can be unsuitable. The exchangeable cations (K^+^, Na^+^, Ca^2+^, and Mg^2+^) that can be replaced if the zeolite is immersed in a solution containing ammonium ions can be measured using a spectrometric method (ICP-OES), and then can be used to calculate the Cation Exchange Capacity (CEC) [49].

**Figure 2:**
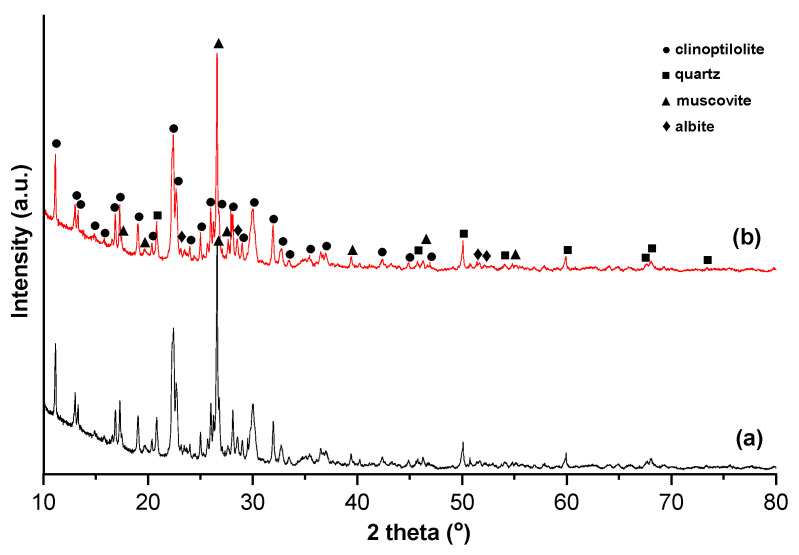
X-ray diffraction patterns of the zeolite samples: (**a**) unmodified natural zeolite and (**b**) silver-exchanged natural zeolite [5].

Zeolites’ mineralogy and structure is achieved using X-ray diffraction (XRD) which identifies crystalline phases and provides information about crystallinity and unit cell parameters. The morphology and size of zeolite crystals can be determined using scanning electron microscopy (SEM) transmission electron microscopy (TEM). Fourier-Transform Infrared Spectroscopy (FTIR) can detect framework vibrations (Si–O, Al–O bonds) and functional groups, confirming zeolite framework and identifying impurities [50]. Brunauer–Emmett–Teller (BET) and porosimetry (N_2_ adsorption–desorption) provides textural properties such as specific surface area, pore size distribution, and pore volume [51]. In silver-exchanged zeolite, the XRD analysis shows no difference in the peak patterns comparing to the unmodified samples, which indicates that the crystal structure of the zeolite was unaffected after silver modification. An example of X-ray diffraction (XRD) analysis of unmodified and silver-exchanged natural zeolites presented by Senila et al. [5] for zeolite prepared for Rn removal from air is displayed in Figure 2.

As indicated in XRD pattern, the natural zeolite sample contained clinoptilolite, muscovite, quartz, and albite, and no additional peak appeared after the Ag loading on the sample.

Silver can be loaded into zeolites via ion exchange, impregnation, or deposition. The location of Ag into zeolite depends on the zeolite Si/Al ratio, pore size and topology, charge of the framework, method of incorporation [52]. There are two possible locations: into the framework/pores (inner sites), exchanged into the cation sites or on the framework outer surface [53]. There are several possibilities to identify the location of Ag into the zeolite. If the measurement of total Ag content using XRF or ICP cannot distinguish its location, if total Ag content ≤ CEC, most likely Ag is inside the framework, in the place of exchangeable cations. On contrary, if total Ag > CEC, Ag deposits exist on the outer surface. When using XRD, no significant changes appear into the spectra if Ag is exchanged into framework pores, whereas establishment of Ag nanoparticles on the outer site can display small metallic Ag reflections. SEM analysis can disclose surface Ag nanoparticles or agglomerates [54].

H-type zeolites contain acidic sites capable of exchanging protons (H^+^) with metal cations such as Ag^+^. During this ion-exchange process, H^+^ ions are replaced by Ag^+^ ions without altering the zeolite’s framework structure. The resulting Ag-exchanged zeolites exhibit unique properties due to the incorporation of Ag^+^ ions and their interaction with the zeolite’s porous network [55]. H-type zeolites are produced under controlled conditions to attain the chosen framework structure and ion-exchange capacity. Recent studies have focused on increasing the performance of silver-exchanged zeolites by optimizing the Ag loading to attain targeted properties without compromising the zeolite’s structure, controlling Ag speciation, and combining Ag-exchanged zeolites with other materials for improved performance [56]. Table 4 provides a comparative overview of the primary advantages and limitations associated with the most common silver exchange techniques.

### Factors Influencing the Properties of Silver-Exchanged Zeolites

The efficacy of silver-exchanged zeolites in applications ranging from catalysis to antimicrobial action does not solely depend on the characteristics of silver, but it is also influenced by the physicochemical characteristics of the zeolite and the method of silver incorporation. Key parameters include the framework topology, the Si/Al ratio, the cation composition and preparation method.

The framework topology determines the size, shape, and connectivity of the pores and cages. This directly controls the accessibility, location, and speciation of the silver. Small-pore zeolite frameworks, such as Linde type A (LTA), primarily confine silver to isolated Ag^+^ ions, making them suitable for controlled ion release. After mild activation only sub-nanometer Ag^+^ clusters are present which exhibit a slow, long-term Ag^+^ leak, thus providing optimal conditions for prolonged antimicrobial action [28,57]. In contrast, large-pore zeolites such as Faujasite (FAU) and Beta (BEA) facilitate the formation and stabilization of oligomeric silver clusters and nanoparticles within their spacious supercages. These confined silver species have been shown to play a crucial role in catalytic oxidation reactions, owing to their enhanced accessibility and redox activity. Furthermore, the presence of large pore openings and a reduced concentration of extra-framework cations promotes the mobility and dispersion of silver ions (Ag^+^) and clusters, thereby improving their reactivity [58].

The Si/Al ratio influences the physicochemical characteristics of silver-exchanged zeolites by controlling the cation exchange capacity, dispersion, speciation and stability. Low-silica zeolites (e.g., Zeolite A) possess a high density of anionic sites, enabling high Ag^+^ loadings and strong ion binding, which is beneficial for sustained antimicrobial release [59]. Conversely, high-silica zeolites (e.g., ZSM-5) feature a lower density of exchange sites and a more hydrophobic character [29]. This weaker electrostatic field facilitates the reduction of Ag^+^ to metallic Ag^0^ nanoparticles during thermal activation, a crucial step for creating active catalysts for hydrocarbon oxidation.

The cation composition influences the properties of silver-exchanged zeolites, including the type, concentration, presence of competing cations, and mobility of silver species [28]. The composition of cations in the solution during the ion-exchange process affects both the antimicrobial activity and structural properties of the zeolite. The presence of other cations alters the ion-exchange equilibrium by reducing the amount of silver incorporated into the zeolite. The type and concentration of exchangeable cations within the zeolite structure, such as Na^+^, K^+^, or Ca^2+^, also affect the zeolite’s capacity to host silver and the mobility of silver within the zeolite framework [60]. Higher concentrations of these cations decrease the amount of silver that can be exchanged into the zeolite structure. Additionally, the presence of complexing reagents influences the silver-exchanged zeolite. Incorporating silver into the zeolite leads to a decrease in the zeolite’s surface area and micropore volume. The preparation method is a crucial factor influencing the properties of silver-exchanged zeolite [28,43]. Parameters such as silver concentration, solution pH, temperature, and distribution of silver incorporated into the zeolite framework influence the performance of silver-exchanged zeolites [28]. The wet/impregnation method, particularly drying can induce partial migration and aggregation of silver whereas calcination at high temperature convert Ag^+^ to silver oxide. Chemical reduction influences the properties of silver exchange zeolite by converting silver ion into metallic silver nanoparticle. This influences pore structure, antimicrobial activity, and structural change [43,61].

## 5. Applications of Silver-Exchange Zeolites

**Figure 3:**
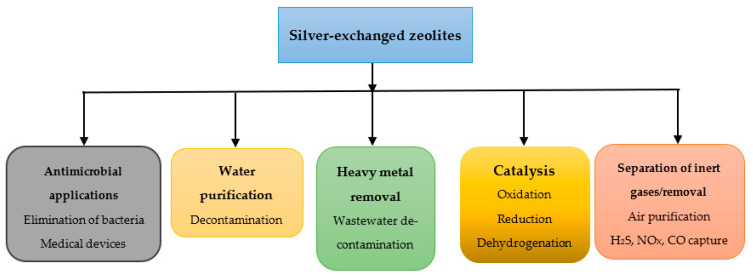
Application of silver-exchanged zeolites.

Figure 3 presents the main applications of silver-exchanged zeolites, which are discussed in detail bellow.

### 5.1. Antimicrobial Applications of Silver Exchanged Zeolites

Silver-exchanged zeolite is used as an antimicrobial additive because it can release silver ions via ion exchange in a controlled manner. Its chemical and thermal stability makes it an effective carrier for silver-based antimicrobials. Its porous structure facilitates the slow release of antimicrobial metals and allows for rapid regeneration. Natural and synthetic zeolites were used for antimicrobial applications. Natural zeolite, specifically ion-exchanged clinoptilolite with Ag^+^, was examined for its antimicrobial activity against *E. coli* [62]. Dutta and Wang [63] reported the same utilization of zeolite as supported silver as antimicrobial agents.

Silver immobilized into zeolite has antimicrobial activity on Gram-negative (e.g., *Acinetobacter*, *Escherichia*, *Pseudomonas*, *Salmonella* and *Vibrio*) and Gram-positive bacteria (e.g., *Bacillus*, *Clostridium*, *Enterococcus*, *Listeria*, *Staphylococcus* and *Streptococcus*), fungi (e.g., *Aspergillus niger*, *Candida albicans*, *Saccharomyces cerevisiae* and *Penicillium citrinum*), virii (e.g., Hepatitis B, and HIV-1). Metals combined with Ag-functionalized zeolite could have antifouling effect and increase the antimicrobial efficiency [64]. *Escherichia coli* bacteria were eliminated by a synthesized silver-zeolite film that used a porous alumina support, as presented by Sabbani et al. [65].

The types of zeolites used for Ag^+^ exchanged are: low Si/Al zeolite (zeolite A, Faujasitic zeolites, EMT), high Si/Al zeolites (mordenite, tritanosilicates (ETS), ZSM-5, zeolite beta), and zeolite membranes. The matrices used for zeolite-silver supports include polymer composites (synthetic polymers such as polyvinylidene fluoride, polyterephthalate, polyurethane, polyamide, polyethylene, polysulfone, polyvinylchloride, polyvinylalcohol, silicone elastomers, and polyether ketone), biopolymers (polylactic acid, natural rubber, alginate—a polysaccharide), textile composites (cellulose, cotton), metal coatings (stainless steel, dental materials), and environmental or consumer materials (odor prevention products, metal door handles, cement/concrete, paper, and food-related applications).

The cations from groups I and II of the periodic table (Na, K, Mg, Ca) situated within the framework of zeolites are mobile and can be exchanged with silver ions (Ag^+^). Silver ions can be adsorbed into the zeolite through ion exchange with the cations present on the zeolite surface. Due to the small size of the silver cation, they can be readily adsorbed. Silver ions have antimicrobial properties, high thermal stability, low volatility, and exhibit cytotoxicity toward animal cells (dependent on silver concentration), being relatively inert and safe. As an antimicrobial agent, silver has two drawbacks: (a) bacteria resistant to silver, and (b) the formation of insoluble precipitates (AgCl, Ag_2_S) that can occur in the reaction between silver ions and zeolite-Ag with various electrolytes (chloride or sulfide anions) in solution. This situation reduces the antimicrobial activity of silver impregnated zeolite. This problem can be addressed by increasing the concentration of silver. Silver ions are less stable in aqueous solutions, but they can be reduced to metallic silver (Ag^0^) in the presence of light and heat. Therefore, incorporating silver ions into a material can mitigate this issue. According to studies in the literature, silver ions exist in their ionic form only within AgY-type zeolite. Silver ions can be released into the solution only if they exchange with other cationic ions present in the solution and only in the presence of bacterial cells. Thus, zeolite can retain silver ions in their ionic form. Compared to other transition metals (e.g., Zn, Cu), silver ions exhibit higher antibacterial activity, and zeolites demonstrate greater selectivity for Ag than for Zn and Cu.

**Figure 4:**
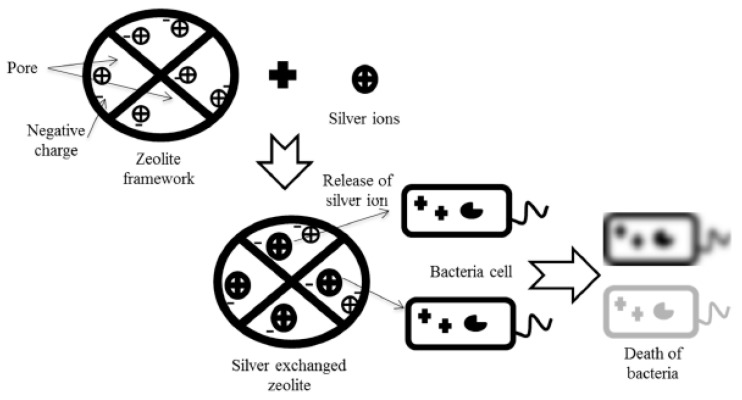
Schematic diagram illustrating the formation of Ag-modified zeolite and the mechanism for its antibacterial activity. Reproduced from Salim & Malek [67].

Figure 4 presents a schematic diagram illustrating the formation of Ag-modified zeolite and the mechanism for its antibacterial activity. Adsorbents such as zeolites, alginate composite, polymer, cellulose nanocrystal, polypropylene, cellulose-graphene oxide nanocomposite, and silica hybrid particles use silver in their composition [66]. In these materials, silver exists in its metallic form (Ag^0^). In medical applications, silver zeolite is combined with other materials, such as sulfadiazin, 3-aminopropyltriethoxysilane (APTES) and other metals (e.g., Zn and Cu), to act as a drug carrier and antibacterial material.

Silver acts as an effective antibacterial agent when functionalized with organic groups prior to exposure to bacteria. Hydroxyapatite is used to enhance its mechanical properties and reduce its coloration, thereby broadening its applicability against a wider spectrum of bacteria.

The first patent on the use of silver-modified zeolites as antibacterial agents was published in 1990 [68]. Nanozeolites have been studied for this purpose. Currently, silver nanozeolites are combined with polyvinyl alcohol (PVA) and polydopamine (PDA) to prevent long-term antifouling. The bacteria commonly studied for antibacterial properties of silver-modified zeolites are *Escherichia coli*, *Staphylococcus aureus*, *Klebsiella pneumoniae*, and *Pseudomonas aeruginosa*. *Escherichia coli* (Gram-negative) bacteria are found in water and food. *Staphylococcus aureus* (Gram-positive) is a common cause of skin infections. *Klebsiella pneumoniae* is responsible for pneumonia, and *Pseudomonas aeruginosa* is resistant to many antibiotics.

Silver zeolite is effective against numerous microorganisms and can kill *E. coli* bacteria even after 12 months of immersion in water and prolonged exposure. The effectiveness of *E. coli* removal depends not only on the silver content but also on the geometry and size of the pores. In their study, Belkhair et al. [69] developed a series of silicone elastomers loaded with silver-zeolite to eliminate *Escherichia coli* and *Staphylococcus epidermidis*. The silver content in the zeolite was 14%. Functionalizing the silver zeolite with silicone improved the composite’s mechanical properties and reduced its color intensity. The addition of elastomers did not significantly affect the antibacterial performance but enhanced the filler-matrix interaction. This composite could be used in the production of medical devices with antibacterial activity [69].

The first study on Ag-exchanged zeolites as biocide agents was reported by Hutson et al. [70], who described the ion exchange process of silver ions into the zeolite structure and demonstrated their antimicrobial properties. Following this study, Cerrillo et al. [71] reported that silver-exchanged zeolites function effectively as bactericidal additives in polymeric materials. Cerrillo et al. [71] reported the synthesis, characterization, and performance of silver-exchanged zeolites as functional additives, focusing on their role in enhancing the hygienic and functional properties of polymeric materials. By incorporating Ag^+^ ions into the porous structure of zeolites, these materials provide controlled and sustained release of silver, which exhibits strong antimicrobial activity against a wide range of bacteria. The zeolite framework stabilizes the silver ions, preventing their aggregation. Additionally, the zeolite enhances the thermal and mechanical stability of the polymer matrix. When incorporated into polymers, Ag^+^-exchanged zeolites inhibit bacterial proliferation on surfaces, suggesting potential applications in medical devices, packaging, and coatings [72].

Ferreira et al. [73] reported the preparation of bimetallic materials based on NaY zeolite using the ion exchange method, employing zeolite Y in its sodium form (NaY) with different metals (Co, Zn, and Ag). The materials were tested for antimicrobial activity in vitro using the bacterium *Escherichia coli* and the yeast *Saccharomyces cerevisiae* as indicator strains. Among all the materials, the one containing Zn/Ag was the most effective for the given purpose [73].

Wu et al. [74] reported a paper related to the impact of silver incorporation into nanocomposite membranes on their antimicrobial properties and longevity. The parameters studied included silver loadings, valence state, surface coating benefits and renderability. The increase in the amount of silver in the membrane increased the antimicrobial activity. Reducing silver ions (Ag^+^) to metallic silver (Ag^0^) stabilized the silver within the membrane, leading to a slower release rate. This modification significantly prolonged the antimicrobial efficacy of the membrane against bacterial growth. The study demonstrated that membranes with silver-zeolite coatings effectively inhibited bacterial attachment and growth on their surfaces, even when bacteria were present in the surrounding suspension. The membranes exhibited the potential for sustained antimicrobial activity, suggesting their suitability for applications requiring long-term biofouling control [74]. Chen et al. [43] reported on the production of silver ion-exchanged zeolite X nanostructures as antibacterial agents for the elimination of methicillin-resistant *Staphylococcus aureus* (MRSA) by using 4–16 ug/mL after 24 h exposure. Efficacy was often highest at relatively low silver concentrations. At low Ag concentration, the ions were dispersed inside the zeolite pores. At high concentration, Ag^+^ can aggregate to Ag_2_O or Ag^0^ [33].

### 5.2. Applications in Water Treatment

Wastewater treatment using zeolites is one of their most well-known applications. The presence of heavy metals (Zn, Cr, Pb, Cd, Cu, Mn, Fe, etc.) in wastewater is a common environmental problem, and their removal using natural zeolites has been extensively studied alongside other technologies such as chemical precipitation, ion exchange, adsorption, membrane filtration, coagulation–flocculation, flotation, and electrochemical methods. Various types of natural zeolites from deposits worldwide have demonstrated strong ion-exchange capacities for cations such as ammonium ions and heavy metals. The modification of natural zeolites can be achieved through several methods, including acid treatment, ion exchange, and surfactant treatment. Modified zeolites also exhibit high adsorption capacities for organic materials and anions. The ion exchange between the zeolite and the heavy metal ions is of adsorption–desorption type, and the process is determined by transfer through the pores of the zeolite. Before being used, zeolites must undergo activation operations by various methods, including thermal methods, chemical methods, or physical treatments. The physical treatment consists of grinding the zeolite and washing it until the very fine powders are removed. The chemical treatment consists of treating the zeolite with acids or bases. The negative charge of zeolites is balanced by interchangeable cations (such as sodium, magnesium, potassium, or calcium). The method consists of replacing the pollutant ion in the wastewater with another less polluting ion, which leads to obtaining a compound that is easier to remove. Zeolite has the best cation exchange capacity among minerals and is capable of selective cation exchange.

Akhigbe et al. [62] reported on the efficiency of silver-modified clinoptilolite for the complete removal of *Escherichia coli* and heavy metals from aqueous solutions. Silver ions are gradually released from the zeolite matrix and interact with bacterial cell walls. Table 5 summarizes the metal selectivity profiles on various types of zeolites, illustrating their differential affinities and ion-exchange capacities.

Pb has a high affinity for ion exchange with most zeolites. The selectivity of ion exchange depends on the ionic concentration and pH level of the solution. Therefore, these factors must be considered in wastewater treatment. The pH of the solution affects the adsorption of metal cations because zeolites are not stable in highly acidic or basic environments. Al dissolves in the zeolite structure under acidic pH conditions, while silicon dissolves under basic pH conditions. Thus, to achieve equilibrium and ensure the adsorption of metal ions, a neutral or slightly acidic pH is preferred. The adsorption characteristics of zeolites are influenced by their physical and chemical properties, including the initial composition of metals in wastewater, pore size, surface area, zeolite dosage, temperature, contact time, and stirring speed. Natural zeolite can adsorb Cu^2+^ ions at a rate of 66.1%, Co^2+^ at 77.9%, Zn^2+^ at 45.9%, and Mn^2+^ at 19.8%. To enhance the adsorption of Mn^2+^ and Sb^3+^ ions, the zeolite is treated with solutions of HNO_3_ and NaOH. Existing selectivity studies have been based on kinetic mechanisms and adsorption isotherm modeling combined with adsorption conditions [67].

Compared with other materials such as Cu, Fe, and TiO_2_, Ag-zeolite is the most efficient for eliminating both pathogens (generally *E. coli*) and metals (Pb^2+^, Cd^2+^, Cu^2+^) [77]. Ag^+^ and Cu^+^ exhibit a high affinity for olefins. Zeolites could potentially substitute the propylene-affinity through cation-exchange. The metals could allow σ and pi interaction with propylene. In the first stage, σ bonds form between the sigma orbitals of the carbon electrons and the empty d or p orbitals of the metal. In the second stage, back-donation occurs from the filled orbitals of the metal to the empty π orbitals of the carbon. The exchange of Ag^+^ ions is easier than that of Cu^+^ ions. The silver exchange zeolite has a good adsorption selectivity for propylene and propane [78].

Fernandes Rocha et al. [26] reported the elimination of *Microcystis aeruginosa* from potable water using zeolite impregnated with silver nanoparticles. *Microcystis aeruginosa* poses risks to aquatic systems, drinking water quality, and public health. The zeolite used was natural zeolite that had been activated and subjected to an ion exchange method to replace zeolite cations with Ag^+^, followed by an impregnation method for the direct deposition of silver nanoparticles (AgNPs) onto the zeolite surface. The new zeolite demonstrated an 80% removal efficiency of *M. aeruginosa* cells [26].

Darmayanti et al. [79] reported a study regarding industrial wastewater treatment by using membrane silver nanoparticles zeolites Na-Y/PVDF. Two types of membranes, thermally induced phase separation (TIPS) and dip coating methods have been employed to modify hollow fiber membrane with silver nanoparticles (AgNPs)-Zeolites. The study demonstrates that the addition of zeolite and silver nanoparticle improved the wastewater purification [79]. The presence of per- and polyfluoroalkyl substances (PFAS) in wastewater is currently a major problem. PFAS are widely generated across many industries, such as textiles and coatings. Their persistence in the environment and their resistance to biodegradation, oxidation, and hydrolysis pose significant concerns contributing to long term risks. Conventional methods, such as activated carbon, ion exchange resins, nanofiltration, reverse osmosis, photocatalysis, and zeolites have their advantages and disadvantages. The zeolites used for PFAS adsorption include all-silicazeolite β, β-zeolite CP811C, CTAB-coatedCP811C, β-zeolite- CP814E, PDADMAC-coated CP814E, zeolite sodiumsilicate composite 1000. Some studies reported the use of 2-β-type zeolite with different Si/Al ratio (25:1 and 300:1) and by using some materials, such as cetyltrimethylammonium bromide (CTAB) or poly(diallyldimethylammonium chloride (PDADMAC). The negative charge of zeolites is unfavorable for the adsorption of short-chain perfluoroalkyl acids (PFAA). Zeolites modified with different materials can improve the PFAS adoptions. The thermally stable structure of zeolites enables their regeneration. β-zeolite has a complete desorption of perfluorooctanoic acid (PFOA) at 360 °C and perfluorooctane sulfonate (PFOS) at 550 °C [80]. Mancinelli et al. [64] reported a method for eliminating PFOS and PFOA from wastewater using silver-FAU-exchanged zeolites with varying silica-to-alumina ratios. Ag-exchanged zeolites had 60% and 32% removal efficiencies for PFOA and PFOS, respectively. The incorporation of Ag not only improves the adsorption efficiency, but may also provide antifouling properties, suggesting a synergistic effect. These results highlight Ag-exchanged Y zeolites as promising multifunctional materials for PFAS remediation in water [64].

### 5.3. Removal of Metals, Mercury, Iodine

Silver-exchanged zeolites have been extensively studied as efficient sorbents for removing metal ions from aqueous solutions because of the strong ion-exchange affinity of Ag^+^ compared to competing cations, as well as the stabilizing effect of the aluminosilicate framework. The aluminum content in the framework determines ionic exchange capacity by influencing the number of negatively charged sites available for charge compensation. In silver-exchanged zeolites, these sites are initially occupied by Ag^+^ cations that can be replaced by other metal ions, such as Pb^2+^, Cd^2+^, Cu^2+^, and Zn^2+^. Capacity depends on the Si/Al ratio and the efficiency of Ag^+^ exchange. Numerous factors influence the kinetics of this exchange process, including diffusion, cation hydration energy, exchange conditions, and the strength of silver binding [81].

When silver-exchanged zeolites are used for metal removal, they can interact in two ways: (a) direct exchange between the metal ions and Ag^+^, or (b) co-adsorption/complexation, where reduced silver species or Ag clusters provide additional binding sites. Generally, the exchange rate is lower for the replacement of Ag^+^ compared to more mobile cations due to the strong interaction between Ag^+^ and the framework. Thermal treatment leads to the formation of clusters and drastically reduces the exchange capacity [81].

Mercury (Hg) is the most toxic element found in aqueous media because it is transformed into methylmercury through biochemical reactions. Mercury is released into the atmosphere by human activities such as combustion, gold production, and the manufacturing of non-ferrous metals and cement. In the atmosphere, mercury is found in elemental form (Hg^0^), as oxidized mercury (Hg^2+^), and as particulate-bound mercury (Hgᵖ). Natural zeolite, both unmodified and modified with Ag^+^, Ag_2_O, and Ag^0^ forms, is used to remove mercury from aqueous solutions of Hg(NO_3_)_2_ and HgCl_2_. The studies reveal that unmodified zeolite with Ag can remove Hg from Hg(NO_3_)_2_ but not from HgCl_2_ solutions. Modification of the zeolite does not significantly influence the removal of mercury from Hg(NO_3_)_2_. The study demonstrates a strong relationship between ion exchange, surface Ag-Hg reactions, and the speciation of Hg in the aqueous phase [8].

Czarna et al. [48] reported the use of fly ash-derived zeolite and silver modified with ash-derived zeolite for mercury removal from water and wastewater generated during wet flue gas desulfurization (FGD) processes [82]. Reske et al. [45] reported the elimination of silver ions from water using zeolite-impregnated graphite oxide (GOIZ) under elevated temperature and pressure conditions in a fixed-bed column. The reaction mechanism involves a redox process in which Ag^+^ adsorb on the surface of Ag^0^, electrostatic interactions occurring between the surface. Additionally, interactions occur between negatively charged Ag^+^ functional groups of GOIZ and cationic-exchange GOIZ, along with cation exchange [45].

Silver-exchanged zeolite Y was used for the removal of volatile methyl iodine (CH_3_I), a compound critical for maintaining nuclear safety in accident scenarios and during spent fuel reprocessing. Silver-exchanged zeolite Y has been extensively studied as a promising adsorbent due to its strong affinity for iodine species. Its effectiveness is strongly influenced by the structural state, dispersion, and dynamic behavior of the silver-zeolite system. Additionally, humidity affects the adsorption strength and binding capacity for CH_3_I [83]. Chebbah et al. [84] reported the production of novel Cu, Ag, and Co ion-exchange ZSM-5 zeolite adsorbent for inorganic stabilizers of nitrocellulose. Nitrocellulose is used in propellants, explosives, and coatings, but its use in practice is limited due its thermal instability and risks during storage and heading. The study showed that incorporated silver into ZSM-5 zeolite stabilizes nitrocellulose by the formation of linkages with nitro groups, delaying the thermal decomposition and could be used as nitrocellulose-based materials in energetic and industrial application [84]. Sadeghi et al. [85] used Ag-clinoptilolite impregnated with tenorite (CuO) nanoparticles (NPs) to remove radioactive strontium-90 (^90^Sr) from water samples. Some studies were conducted on the effects of pH, the amount of adsorbent, and contact time, and the optimal conditions were obtained after six hours of contact, yielding 97% [85].

In a study conducted by Elmekawy et al. [86], silver-modified zeolites (13X-Ag, 5A-Ag, chabazite-Ag, and clinoptilolite-Ag) were synthesized and evaluated as adsorbents for iodine removal under environmentally relevant conditions. Among these, chabazite-Ag demonstrated the highest adsorption capacity, followed by 13X-Ag and 5A-Ag, while clinoptilolite-Ag exhibited the lowest capacity. Kinetic modeling indicated that iodine adsorption follows a pseudo-second-order model, consistent with chemisorption-driven mechanisms. This model suggests that chemisorption, rather than simple physical sorption, governs iodine uptake on silver-exchanged zeolites [86].

### 5.4. Applications in Separation of Inert Gases/Adsorption

Xenon is used in lighting and optics, medicine (as an inhalation anesthetic), aerospace industry (ion propulsion for satellites), research technology (xenon lasers and particle detectors), and other applications such as cryogenics and gas thrusters in scientific experiments. The production of pure xenon is associated with substantial costs. An alternative method for separating xenon is the use of an adsorbent at nuclear fuel reprocessing facilities. Silver exchange zeolite was used for the separation of xenon directly from the air. Gueibe et al. [87] reported a method that uses silver-exchange zeolite for the separation of xenon from the air [87]. Heinitz et al. [88] reported the adsorption of radioactive noble gas radon (^222^Rn) at room temperature using silver-modified Engelhard Titano-Silicate zeolites (Ag-ETS-10) and Ag-ZSM-5 (Zeolite Socony Mobil, ZSM). Factors affecting radon adsorption include water vapor and the carrier gas. These new materials can adsorb ^222^Rn without the need for cryogenic cooling [88].

Radioxenon isotopes released during fission-based medical isotope production present significant challenges for environmental safety and regulatory compliance. Silver-exchanged zeolites provide a promising solution for their capture and mitigation due to their high cation-exchange capacity, thermal stability, and the strong affinity of Ag^+^ ions for xenon species. Gueibe et al. [87] reported the potential of silver-exchanged zeolites as efficient sorbents for radioxenon mitigation in medical isotope production facilities, contributing to both operational safety and environmental protection [87]. Van Zandvoort et al. [89] reported a study on the use of CaA and AgA zeolites for the adsorption of olefins from gas mixtures (N_2_, H_2_, CH_4_, C_2_H_4_, C_2_H_6_, CO, and CO_2_) resulting from biomass gasification. Zeolites exhibit specific adsorption of ethylene based on cation size, polarizability, and the interaction between the π-bond and the olefin. The study showed that CaA zeolite has the highest adsorption capacity for ethylene under repeated regeneration, while AgA zeolite exhibit higher adsorption and high ethylene recovery. However, a drawback of AgA zeolite is the reduction of Ag^+^ to metallic Ag and the agglomeration of larger particulates due to the presence of gases [89]. Xv et al. [90] explored the luminescence behavior of silver nanoclusters encapsulated in faujasite (FAU) zeolite and demonstrated how framework modifications influence their emission characteristics. By systematically varying the Si/Al ratio, cation distribution, and extra-framework composition, the local environment of the confined Ag species was tailored to optimize their optical response. Spectroscopic analyses revealed that higher framework silica content enhances emission stability by reducing non-radiative decay pathways, while controlled cation exchange fine-tunes the excitation–emission profiles. The study showed that faujasite zeolite serves two functions: as a confining host and as a chemically modified material that imparts desirable properties to silver clusters for applications in sensing, light-emitting devices, and photocatalysis [90].

Another application of silver-zeolite is in the petrochemical industry for the separation of olefins from paraffins [91]. The interaction between alkenes and transition metal cations located within the channels and cavities of microporous zeolites enhances the separation efficiency [92]. Ethylene is an important chemical with numerous applications. It functions as a plant hormone that regulates the growth of fruits and vegetables, as well as maintaining their freshness under controlled conditions of humidity, temperature, and carbon dioxide. Ethylene is released at high humidity. Ren et al. [93] reported using a silver-modified form of silicalite-1 (from the ZSM-5 family) to capture trace amounts of ethylene under humid conditions. Silicalite-1 has low affinity for ethylene, but the impregnation with Ag enhances ethylene adsorption at high relative humidity through II-complexation of Ag^+^ with the double bond in ethylene [93]. Liu et al. [94] reported a study regarding the use of Ag-Cu modified ZSM-5 zeolite for the elimination of NO_X_ and ammonia from coal-fired flue gas. Nitrogen oxides are one of the most prevalent pollutants generated by various sources and they can lead to numerous environmental issues including greenhouse effect, acid rain and photochemical smog. Currently, the technology used for NO_X_ elimination is catalytic reduction using catalysts.

Another utilization of silver exchanged zeolites is to obtain biofilm that combats dental caries. The following cariogenic pathogens were selected: *Streptococcus sanguinis*, *Streptococcus mutans* and *Streptococcus gordonii*, which form single-species biofilms. The results shows that Ag containing zeolite have better antibacterial properties. Usalamin et al. [95] reported the use of zeolite modified with gallium, zinc, and silver for supporting the production of benzene, toluene, and xylene (BTEX) through the aromatization of ethylene on HZSM-5 type catalysts modified with metals. The metal species are responsible for catalyzing the dehydrogenation pathways, with gallium being the most effective for BTEX production. Increasing the temperature and the partial pressure of ethylene favors the aromatization of ethylene. BTEX are used for the production of plastics and fine chemicals. Currently, BTEX are obtained by naphtha cracking to light olefins [95].

### 5.5. Catalysis Reactions

Rashidvand et al. [53] employed silver-modified ZSM-5 zeolite as a catalyst in the petroleum waste cracking process, achieving a yield of 9.78%. The zeolite was prepared using a microwave-assisted method. The process involved mixing two solutions for 4 h at 100 °C: (a) orthosilicate, ethanol, and sodium hydroxide were mixed at 100 °C for 2 h; (b) tetrapropylammonium hydroxide was combined with aluminum nitrate and sodium hydroxide solution [53].

Mokhtar et al. [96] reported a study regarding a synthesized bimetallic Ag-Ni doped LTA (Linde Type A) zeolite from kaolin as a multifunctional catalyst. The structural characterization confirmed the successful incorporation of Ag and Ni into the LTA framework, enhancing the catalytic efficiency and antimicrobial activity. The material exhibited remarkable dual functionality: efficient degradation of Congo red dyes through catalytic oxidation and strong antibacterial activity for *Klebsiella pneumoniae* and *Enterococcus*. The synergistic effect of Ag and Ni doping significantly improved the performance compared to monometallic or undoped zeolites. These findings suggest that Ag–Ni/LTA zeolite is a promising, sustainable material for integrated wastewater treatment and microbial disinfection [96].

Another use for zeolite is as a catalyst for the production of 1,3-butadiene in the following stages: the formation of acetaldehyde from ethanol; aldol condensation to crotonaldehyde; Meerwein–Ponndorf–Verley (MPV) reduction of crotonaldehyde with ethanol to crotyl alcohol and acetaldehyde (AA); and the dehydration of crotyl alcohol to 1,3-butadiene. Tantalum-containing SiBEA zeolite with an isolated mononuclear Ta(V) framework doped with Ag, Cu, and Zn was used as a catalyst for obtaining 1,3-butadiene. Using zeolite doped with various metals decreases ethanol conversion in the following order: TaSiBEA < ZnTaSiBEA < AgTaSiBEA < CuTaSiBEA [97]. These findings were consistent with those reported in other studies [98,99,100,101].

Zheng et al. [102] reported the use of silver-exchange zeolite Y to catalyze a selective insertion of carbene transfer reaction. Simple counter-cation exchange within the zeolite framework modulates both activity and selectivity, with the zeolite acting as a macroligand to fine-tune the silver active sites. This recyclable solid catalyst enables in situ carbene generation from diazoacetate and their selective insertion into C–H (e.g., cyclohexane) and C–O (e.g., water) bonds at silver loadings as low as ≤0.1 mol%. Moreover, the reaction with adsorbed water facilitates deep drying of the HY zeolite, highlighting a dual role in catalysis and framework modification [102].

## 6. Advantages and Limitations

The advantages and limitations associated with silver-exchanged zeolite, highlighting their potential applications, as well as the challenges impacting their performance are summarized in Table 6.

## 7. Challenges and Future Perspectives

Silver exchanged zeolites have several critical challenges that limit their efficiency and practical applicability. These challenges include silver leaching, cost, thermal and chemical stability, selectivity of catalysis, environmental and health concerns, and regeneration and reusability. The recovery of silver after the leaching process depends on the reagent used, its toxicity, any additional pretreatment, and environmental pollution. The leaching processes are divided into two categories: (i) converting silver to chloride and dissolving it, and (ii) converting silver to a salt in water. For antimicrobial applications, silver leaching (ionic or nanoparticle) increases toxicity and reduces longevity. Future approaches will address the use of endocapsulation or surface treatments to extend the material’s lifetime.

The high cost of silver compared to other metals presents challenges for its industrial use. The main challenges are developing new low-cost synthesis routes for silver-exchanged zeolites, optimizing ion exchange processes, and partially substituting silver with less expensive metals. Future research should focus on developing advanced materials that minimize pressure drop while enhancing mass transfer and gas adsorption capacity. This approach would improve the overall performance in practical applications. A promising future perspective involves the development of a procedure that uses zeolite to reduce silver leaching and incorporates other metals (e.g., Cu and Zn) to enhance stability and reduce silver loading. The use of a green chemistry approach will be the future perspective in synthesis route.

Further research should be conducted on the integration of nanomaterials and advanced materials, such as polymers, membranes, and composites, to improve the properties of silver-exchanged zeolites and extend their adsorption application. Currently, zeolites are combined with porous compounds such as crystalline cobalt/amorphous LaCoOx [103], hybrid nanoparticles, hydrated copper pyrophosphate ultrathin nanosheets, g-C3N4 (B–CN) as novel 2D bubble-like structures [104], degree-of-sulfurization (DoS) CO-based nanocatalysts, cerium-incorporated Co-based catalysts in nitrogen-doped structures [105], flexible polystyrene/graphene (PS/GR) metacomposites [106], carbon nanotube (CNT)/epoxy composites [107], and mono-phase ceramics of indium tin oxides [53]. In addition to silver, other metals can also be incorporated into the structure of zeolites. Therefore, the development of new catalysts with improved properties represents another challenge, particularly for specific reactions. In the case of noble gas capture (Xe, radon), excellent results were obtained under laboratory conditions. However, the scalability and reproducibility of these processes at industrial level must be validated.

## 8. Conclusions

The review summarizes recent studies on the synthesis, characterization, and applications of silver-exchanged zeolites. These materials are used in antimicrobial treatments, water purification, heavy metal removal, catalysis, and the separation of inert gases. The discussion includes both natural and synthetic zeolites, with particular emphasis on the silver exchange process. The unique performance of these materials stems from the ability of zeolite frameworks to stabilize silver in various forms, ranging from isolated Ag^+^ ions to ultrasmall clusters and nanoparticles.

The challenge remains to control the quantity of Ag to prevent leaching. In antimicrobial applications, Ag-exchanged zeolites have demonstrated remarkable adsorption capacities against various bacteria, such as *E. coli*, *Staphylococcus aureus*, *Klebsiella pneumoniae*, *Pseudomonas aeruginosa*, and *Staphylococcus epidermidis*, etc. In water treatment, silver-exchanged zeolites exhibit a strong capacity to adsorb heavy metals, particularly Cu, Zn, Fe and Pb. Additionally, silver-exchanged zeolites have a high affinity for toxic metal ions and volatile species, especially mercury and iodine. This is attributed to the ion-exchangeable Ag^+^ sites and the strong interaction between silver and donor atoms, such as iodine. Studies show that Ag-zeolites can immobilize Hg^2+^ and elemental Hg, forming stable Ag-Hg compounds within the zeolite matrix. The removal capacity depends on factors such as silver loading, pH, temperature, and the presence of competing ions. Furthermore, silver-exchanged zeolites exhibit a high selective adsorption capacity for inert gases, particularly xenon and radon, due to the strong interaction between Ag^+^ cations and the polarizable ions of these gases. This property makes them excellent materials for applications in nuclear safety and environmental monitoring.

In conclusion, silver-exchanged zeolite is a highly promising material for a wide range of applications and could play a crucial role in next-generation clean energy solutions, environmental remediation, and sensing technologies.

## Figures and Tables

**Table 1:** Structure and chemical formulas of several natural zeolites [22].

Zeolite	Chemical Formula	Structure	Used
Clinoptilolite	(K_2_,Na_2_,Ca)_3_Al_6_Si_30_O_72_·21H_2_O	HEU	Environmental remediation and ion exchange
Mordenite	(Na_2_,Ca)_4_Al_8_Si_40_O_96_·28H_2_O	MOR	Strong adsorption properties
Chabazite	(Ca,Na_2_,K_2_)_2_Al_4_Si_8_O_24_·12H_2_O	CHA	CO_2_ capture and gas separation
Heulandite	(Ca,Na)_2_-_3_Al_3_(Al,Si)_2_Si_13_O_36_·12H_2_O	HEU	Catalysis
Phillipsite	K_2_(Ca,Na_2_)_2_Al_8_Si_10_O_32_·12H_2_O	PHI	Wastewater treatment
Scolecite	Ca_4_Al_8_Si_12_O_40_·12H_2_O	NAT	Ion exchange and adsorption
Stilbite	Na_2_Ca_4_Al_10_Si_26_O_72_·30H_2_O	STI	Catalysis and gas separation
Analcime	Na_16_Al_16_Si_32_O_96_·16H_2_O	ANA	Ceramics and as a molecular sieve
Laumontite	Ca_4_Al_8_S_16_O_48_·16H_2_O	LAU	Hydration behavior

**Table 2:** Comparative molecular formulas, structures, and properties of selected zeolites.

Zeolite	Framework Type	Idealized Molecular Formula	Typical Si/Al Ratio	Pore Opening
Natural zeolite	HEU	(Na,K,Ca)_6_[Al_6_Si_30_O_72_] · 20H_2_O	4–5 (Si/Al high)	3.5 × 7.6 Å & 4.6 × 3.1 Å (10-ring channels)
A (Zeolite A)	LTA	Na_12_[Al_12_Si_12_O_48_] · 27H_2_O	1.0	4.1 Å × 4.1 Å (8-ring)
X (Zeolite X)	FAU	Na_86_[Al_86_Si_106_O_384_] · 264H_2_O	1.2–1.5	7.4 Å (12-ring)
Y (Zeolite Y)	FAU	Na_56_[Al_56_Si_136_O_384_] · 250H_2_O	2.5–3.0	7.4 Å (12-ring)
ZSM-5	MFI	Na_n_[Al_n_Si_96 − n_O_192_] · 16H_2_O	10–1000+	5.1 × 5.5 Å & 5.3 × 5.6 Å (10-ring channels)

**Table 3:** Comparison between natural and synthetic zeolites.

Aspect	Natural Zeolites	Synthetic Zeolites
Genesis	Formed naturally from volcanic/sedimentary processes.	Manufactured hydrothermally under controlled lab/industrial conditions.
Purity	Contain impurities (clays, quartz, feldspar).	High purity, no gangue minerals.
Composition	Fixed, limited Si/Al ratios.	Tunable Si/Al ratios
Structure Variety	~50 species, limited pore variety	>200 synthetic zeolite frameworks
Pore Size & Shape	Limited range; often irregular due to impurities.	Precisely controlled pore dimensions (molecular sieving at Å-scale).
Cation Exchange Capacity (CEC)	Moderate, varies with deposit.	Higher and adjustable
Reproducibility	Quality varies between deposits.	Highly reproducible, batch to batch.
Availability	Abundant, easily mined.	Requires industrial synthesis
Main Applications	Bulk uses: wastewater treatment, soil amendments, animal feed, gas absorption, odor control.	High-tech uses: petrochemical cracking catalysts, detergent builders, molecular sieves, gas separation, fine chemical synthesis.
Cost	Low (mined, minimally processed).	Higher (manufactured under controlled conditions).

**Table 4:** Advantages and drawbacks of different methods used for silver exchange preparation.

Method	Principle	Advantages	Disadvantages
Ion exchange	Replacement of native cations (Na^+^, K^+^, Ca^2+^, etc.) in zeolite with Ag^+^ from aqueous solution	Control of Ag content High stability of ionic silver Uniform dispersion of Ag^+^ at framework sites Maintains crystallinity & pore structure Precise control over Ag loading via exchange conditions	Capacity limited by framework Al content Time-consuming (often multiple cycles) Sensitive to pH/ionic strength (risk of Ag precipitation) Strong Ag binding may hinder later reduction
Wet impregnation	Impregnation of zeolite pores with Ag salt solution, followed by drying/calcination	Simple, fast, and scalable Allows higher Ag loadings beyond cation exchange capacity Suitable for nanoparticle formation after calcination	Often non-uniform Ag distribution Risk of surface deposition & pore blockage Silver aggregation/sintering at high temperature Less control of Ag oxidation state
Chemical reduction	Introduction of Ag^+^ (via exchange/impregnation) followed by chemical reduction (e.g., NaBH_4_, H_2_)	Enables formation of Ag^0^ nanoparticles or small Ag_n_ clusters inside pores Size/dispersion tunable via reduction conditions Zeolite stabilizes clusters against aggregation	Risk of external nanoparticle deposition Non-uniform size distribution Excessive reduction may block pores or destabilize framework Requires careful handling of reductants

**Table 5:** Selectivity of different types of zeolites for metals [22,75,76].

Zeolite	Selectivity	Molar Ratio Si/Al
clinoptilolite	Pb^2+^ > Ag^+^ > Cd^2+^ ~ Zn^2+^ > Cu^2+^	2.7–5.3
clinoptilolite	Pb^2+^ > Zn^2+^ > Cu^2+^ ~ Ni^2+^	4.9
clinoptilolite	Pb^2+^ > Cd^2+^ > Zn^2+^ ~ Cu^2+^	4.2
clinoptilolite	Pb^2+^ > Cd^2+^ > Cu^2+^ > Co^2+^ > Cr^3+^ > Zn^2+^ > Ni^2+^ > Hg^2+^	-
phillipsite	Pb^2+^ > Cd^2+^ > Zn^2+^ > Co^2+^	2.4–2.7
mordenite	Mn^2+^ > Cu^2+^ > Co^2+^ ~ Zn^2+^ >Ni^2+^	4.4–5.5
scolecite	Cu^2+^ > Zn^2+^ > Pb^2+^ > Ni^2+^ > Co^2+^ > Cd^2+^	1.56
chabazite	Pb^2+^ > Cd^2+^ > Cu^2+^ > Zn^2+^ > Co^2+^	2.2–2.6

**Table 6:** Advantages and disadvantages of silver-exchanged zeolite.

Application	Advantages	Disadvantages
Water treatment	Strong adsorption of heavy metals (Pb^2+^, Cu^2+^, Cd^2+^) and organic pollutants. Can be used in existing water treatment systems.	Risk of Ag^+^ to leaching into water Limited regeneration in aqueous system Adsorption can be reduced by fouling from natural organic matter. Repeated use may lead to silver nanoparticle agglomeration, reducing efficiency. Silver significantly increases the overall cost.
Antimicrobial	Sustained antimicrobial action due to gradual release of Ag^+^ ions. Broad-spectrum activity against bacteria, fungi, algae, and viruses. High capability of Ag against various bacterial strains Reduces need for harsh chemical disinfectants. Effective at low concentrations, reducing frequent dosing needs.	Potential cytotoxicity with prolonged exposure. Regulatory concerns over silver release. Expensive compared to other antimicrobial agents. Over time, Ag^+^ release rate may decline, reducing long-term effectiveness. Silver particles may agglomerate, lowering antimicrobial efficiency. Potential cytotoxicity at higher silver concentrations.
Catalysis	Active sites for oxidation, hydrogenation and dehydrogenation reactions. Synergistic effects of Ag with zeolite acidity. Useful in environmental catalysis (e.g., VOC oxidation). Catalyst can be regenerated by calcination or reduction treatments. Can replace or reduce use of more expensive noble metals (e.g., Pt, Pd).	Deactivation due to silver sintering or leaching. Requires precise silver loading and dispersion. More expensive than non-precious metal catalysts. Ag nanoparticles can sinter at high temperatures, reducing activity. Selectivity may decrease if silver migrates or agglomerates. Silver still adds significant cost compared to base-metal catalysts.
Air purification	Strong affinity for halogens (e.g., iodine, bromine) and sulfur compounds (H_2_S, SO_2_). Effective in removing volatile organic compounds (VOCs) and toxic gases. Can operate under a wide temperature range.	Humidity reduces adsorption efficiency. Silver nanoparticles may sinter under high temperatures. Costly compared to activated carbon.
Nuclear safety	Strong affinity for I_2_ and CH_3_I through Ag–I bond formation for iodine capture High thermal and radiation stability of zeolite framework. Effective even at low iodine concentrations.	Silver restructuring reduces long-term performance. High cost due to silver content. Disposal of radioactive Ag–zeolite waste is challenging.

## Data Availability

No new data were created or analyzed in this study. Data sharing is not applicable to this article.

## Abbreviations

The following abbreviations are used in this manuscript:

AgNPs	Silver nanoparticles
Ag^+^	Silver ions
PDADMAC	Poly(diallyldimethylammonium chloride
APTES	3-aminopropyltriethoxysilane
ZSM-5	Zeolite Socony mobil-5
CEC	Cation exchange capacity
STI	Stilbite
ETS	Tritanosilicates
PVA	Polyvinyl alcohol
PDA	Polydopamine
Ag	Silver
Ag^0^	Metallic silver
MRSA	Methicillin-Resistant *Staphylococcus aureus*
TIPS	Thermally induced phase separation
PFAS	Per- and polyfluoroalkyl substances
CTAB	Cetyltrimethylammonium bromide
BTEX	Benzene, toluene, and xylene
PFAA	Perfluoroalkyl acids
GOIZ	Zeolite-impregnated graphite oxide
PFOA	Perfluorooctanoic acid
PFOS	Perfluorooctane sulfonate
Hg	Mercury
Hg^2+^	Oxidized mercury
Hgᵖ	Particulate-bound mercury
NP	Nanoparticles
CH_3_I	Volatile methyl iodine
DoS	Degree-of-sulfurization
FAU	Faujasite
FGD	Flue gas desulfurization
AA	Acetaldehyde
MPV	Meerwein–Ponndorf–Verley
VOCs	Volatile organic compounds
PS/GR	Polystyrene/graphene
CNT	Carbon nanotube
